# Granzyme K Activates Protease-Activated Receptor-1

**DOI:** 10.1371/journal.pone.0021484

**Published:** 2011-06-30

**Authors:** Dawn M. Cooper, Dmitri V. Pechkovsky, Tillie L. Hackett, Darryl A. Knight, David J. Granville

**Affiliations:** 1 Institute for Heart and Lung Health, St. Paul's Hospital, Vancouver, British Columbia, Canada; 2 Department of Pathology and Laboratory Medicine, University of British Columbia, Vancouver, British Columbia, Canada; 3 Department of Anesthesiology, Pharmacology and Therapeutics, University of British Columbia, Vancouver, British Columbia, Canada; University of Brescia, Italy

## Abstract

Granzyme K (GrK) is a trypsin-like serine protease that is elevated in patients with sepsis and acute lung inflammation. While GrK was originally believed to function exclusively as a pro-apoptotic protease, recent studies now suggest that GrK may possess other non-cytotoxic functions. In the context of acute lung inflammation, we hypothesized that GrK induces pro-inflammatory cytokine release through the activation of protease-activated receptors. The direct effect of extracellular GrK on PAR activation, intracellular signaling and cytokine was assessed using cultured human lung fibroblasts. Extracellular GrK induced secretion of IL-6, IL-8 and MCP-1 in a dose- and time-dependent manner in lung fibroblasts. Heat-inactivated GrK did not induce cytokine release indicating that protease activity is required. Furthermore, GrK induced activation of both the ERK1/2 and p38 MAP kinase signaling pathways, and significantly increased fibroblast proliferation. Inhibition of ERK1/2 abrogated the GrK-mediated cytokine release. Through the use of PAR-1 and PAR-2 neutralizing antibodies, it was determined that PAR-1 is essential for GrK-induced IL-6, IL-8 and MCP-1 release. In summary, extracellular GrK is capable of activating PAR-1 and inducing fibroblast cytokine secretion and proliferation.

## Introduction

Granzymes (granule-secreted enzymes) are a family of serine proteases that were once thought to function exclusively in immune-mediated target cell death through a perforin-dependent mechanism. In humans, there are 5 granzymes that differ in both function and substrate specificity: Granzyme A (GrA; tryptase), Granzyme B (GrB; aspartase), Granzyme H (GrH; chymase), Granzyme K (GrK; tryptase) and Granzyme M (GrM; metase). Despite their initial discovery and prediction to act as both intracellular and extracellular proteases, traditional views have limited granzyme function to the intracellular, perforin-dependent induction of cell death. However, over the past few years, evidence has emerged to challenge this view and strongly implies that granzymes exert other non-cytotoxic roles in health and disease [1], [2], [3], [4], [5], [6], [7], [8]

Elevated levels of GrA, GrB and GrK are observed in a wide array of inflammatory diseases such as atherosclerosis, arthritis, chronic obstructive pulmonary disease (COPD), idiopathic pulmonary fibrosis (IPF), and asthma (reviewed in [4], [9]). However, while several studies have focused on the perforin-independent functions of GrA and GrB, little is known concerning the function of extracellular GrK [10], [11], [12], [13], [14]. GrK is found at low levels in the plasma of healthy patients but is markedly elevated in the plasma of patients suffering from viral infections and sepsis [11], [15]. GrK is also elevated in the bronchoalveolar lavage (BAL) fluid of patients suffering from allergic asthma and viral pneumonias [10], [11]. Although our understanding of the proteolytic regulation of extracellular GrK remains poorly understood, recent studies have identified inter-alpha inhibitor proteins (IAIP) as physiological inhibitors of GrK and have shown that a reduction in plasma IAIP levels and free, unbound GrK correspond to increased disease severity [16], [17].

GrK is a highly cationic protease that displays tryptase-like activity, which cleaves after the basic amino acids Lys or Arg and is most closely related to GrA [18], [19], [20], [21]. Despite sharing many substrates with GrA, proteomic profiling has demonstrated that GrK can target a unique set of substrates suggesting it likely functions distinctly from that of GrA [19], [22]. GrA, also a tryptase-like protease, is capable of inducing cell detachment, cytokine release, neurite retraction and activation of Protease-Activated Receptor (PARs) [8], [23], [24], [25]. PARs are a family of G-protein coupled receptors (GCPRs) that mediate the physiological responses to serine proteases (reviewed in [26], [27]). PARs share a unique mechanism of activation that involves the cleavage of an N-terminal extracellular domain which leads to the unmasking of a tethered ligand that, in turn, activates the receptor by intramolecular binding followed by intracellular signaling [27], [28]. PAR-1 is activated by thrombin and trypsin, PAR-2 is a receptor for trypsin and mast cell tryptase, and PAR-3 and PAR-4 are receptors for thrombin [27], [28], [29].

In the present study, we investigated whether extracellular GrK could induce PAR activation in human lung fibroblasts. GrK induced the production of interleukin-6 (IL-6), IL-8 (CXCL8) and monocyte chemotactic protein-1 (MCP-1)/chemokine c-c motif ligand 2 (CCL2) in human lung fibroblasts through the activation of PAR-1. In addition, GrK induced fibroblast proliferation in a PAR-1-dependent manner suggesting that elevated extracellular GrK could augment inflammation and play a role in airway remodeling through the activation of PAR-1.

## Materials and Methods

### Reagents

Cell culture medium Dulbecco's modified Eagle's medium (DMEM), fetal bovine serum (FBS), and PBS were obtained from Invitrogen (Carlsbad, CA, USA). Thrombin, ERK1/2 inhibitor U0126 and the p38 MAPK inhibitor SB202190, and the antibiotics (penicillin and streptomycin) were obtained from Sigma (St. Loius, MO, USA). Granzyme K was obtained from Axxora (Burlington, ON Canada), and the PAR-1 (ATAP-2 cat no: sc-13503) and PAR-2 (Sam11L cat no: sc-13504) neutralizing antibodies were obtained from Santa Cruz Biotechnologies (Santa Cruz, CA, USA). Mouse IgG (Abcam cat no 37355) was used as an isotype control. Antibodies: Phospho-p44/42 (Cell Signaling Technology (clone E10) cat no: 9106s), total p44/42 (Cell Signaling Technology (clone 137F5) cat no: 4695), phospho-p38 MAPK (Cell Signaling Technology (clone 28B10) cat no: 9216), total p38 MAPK (Cell Signaling Technology cat no: 9212s) β-tubulin (Millipore (clone AA2) cat no: 05-661) was purchased from Millipore, goat anti-mouse IRD 700 and goat IRD anti-rabbit 800 were purchased from LI-COR Biotechnology (Lincoln, Nebraska). IL-6, IL-8 and MCP-1 Duoset ELISAs were purchased from R&D Technologies (Burlington, ON, Canada). Dimethyl sulphoxide (DMSO) was purchased from Sigma (St. Loius, MO, USA) and sodium azide was purchased from sigma (St. Louis, MO, USA). Cell lysis solution, protease inhibitor cocktail and phosphatase inhibitor cocktail were purchased from Sigma (St. Loius, MO, USA). BioRad protein reagent and nitrocellulose membranes were purchased from Bio-Rad (Mississauga, ON, Canada).

### Cell Culture

Human fetal lung fibroblasts (HFL) were purchased from ATTC (Cat. No CCL-153). Cells were maintained in DMEM supplemented with 10% (vol/vol) FBS, 100 µg/ml penicillin, 100 µg/ml streptomycin, and kept in a humidified atmosphere with 5% CO_2_. All experiments were performed between passages 5–11. Cells were routinely passaged every 4–5 d. For all experiments, cells were grown to confluency and serum-starved in DMEM with 0.1% FBS for 24 h before treatment.

### Detection of IL-6, IL-8, MCP-1

IL-6, IL-8 and MCP-1 levels were determined using human IL-6, IL-8 or MCP-1 ELISAs, according to according to manufacturer's protocols. Briefly, cells were seeded in 12-well culture dishes (1×10^6^ per well), grown to 90% confluency and serum-starved in DMEM (0.01% FBS) over-night. Cells were then incubated with GrK (10–300 nM), in the absence of any delivery/cell permeabilizing agent (ie. perforin, streptolysin O), or Thrombin (2.5 U/ml), at the concentrations and time points indicated for each experiment in DMEM containing 1% FBS. Supernatants were collected for analysis by ELISA and the remaining cells were trypsinized and counted using a hemocytometer. For inhibitor studies, cells were pre-treated with the ERK inhibitor (U0126; 10 µM) or the p38 MAPK inhibitor (SB202190; 10 µM) or neutralizing antibodies for 45 min prior to stimulation with GrK (200 nM) or thrombin (2.5 U). Cells treated with vehicle alone, heat-inactivated GrK (100 nM, heat-inactivated at 85°C for 5 min) or mouse IgG (5 µg/ml) isotype served as baseline controls.

### Western blot analysis of signal transduction pathways in HFLs upon GrK stimulation

HFL were seeded in 6-well culture dishes at 2×10^6^ cells/well, grown to desired confluency, serum-starved over night and then treated with 10 µM U0126 or 10 µM SB202910 for 45 min prior to treatment with 200 nM of GrK or 2.5 U/ml of Thrombin. After 10 min, 30 min, and 2 h, supernatants were collected for IL-6, IL-8 and MCP-1 determination and remaining cells harvested for Western blot analysis of the levels of the downstream products for phospho-p44/42 (ERK1/2) and phospho-p38 MAPK.

Briefly, cells were rinsed with ice-cold Dulbecco's phosphate-buffered saline (dPBS) and lysed in 100 µl of cell lysis buffer containing proteinase inhibitor cocktail and phosphatase-inhibitor cocktail followed by scraping with a cell scraper. Cell debris was removed by centrifugation (12,000× *g* for 15 min) and protein was quantified by the Bradford method (BioRad) using bovine serum albumin as standard. Samples containing equal amounts of total cell protein were separated by 10% SDS-PAGE, and transferred onto a nitrocellulose membrane using the Bio-Rad wet transfer system at 100 v for 1 h. The membranes were blocked with 2.5% skim milk in TBST (50 mM Tris, pH 7.6, 0.15 mM NaCl, 0.1% Tween 20) for 1 h at room temperature. Membranes were then incubated with antibodies against phospho-p44/42 MAPK (1∶2000), total p44/42 MAPK (1∶1000), phospho-p38 MAPK (1∶2000), p38 MAPK (1∶1000) over night at 4°C with gentle shaking. After several washes in TBST, membranes were incubated with goat anti-mouse IRD 700 and goat IRD anti-rabbit 800 for 1 h at room temperature. Flourescent signal was imaged using the Li-COR Odyssey Infrared imaging system (Li-COR biosciences). Densitometry was used to quantify all bands. Membranes were then re-probed with mAb β-tubulin (1∶5000) for 1 h at room temperature and incubated with goat anti-mouse IRD 800 for 45 min. The relative levels of phospho-p44/42 MAPK or phospho-p38MAPK are expressed as the ratio to β- tubulin.

### PAR-1 desensitization

For PAR-1 receptor desensitization studies, cells were pre-treated with 2.5 U/ml of thrombin for 10 min. Media was removed and cells were washed three times with PBS. For IL-6, IL-8 and MCP-1 measurements, fresh media containing GrK (200 nM) or thrombin (2.5 U/ml) was then added cells for a 24 h incubation period and supernatants were collected and screened as described above. In addition to measuring potential changes in cytokine and chemokine production, thrombin-induced PAR-1 activation will induce ERK1/2 phosphorylation. Therefore, we screened for changes in p44/42 (ERK1/2) activation following receptor de-sensitization in cells were pre-treated with thrombin. Here cells were pre-treated with thrombin (2.5 U/ml) for 10 min, media was removed and cells were washed three times with PBS. Cells were then treated with either GrK (200 nM) or thrombin (2.5 U/ml) for 10 min. Cell lysates were then collected as described above and screened for phospho-p44/42 activation as described.

### Cell viability and proliferation

As extracellular GrA and GrB have been shown to induce cell detachment, we examined the impact of extracellular GrK on cell viability and proliferation in HFLs. Cell viability was measured using the WST-1 assay (Roche, Ltd. Missasauga, ON, Canada) according to manufacturer's instructions. Here, 96-well culture dishes were seeded with 1×10^4^ cells and incubated with media alone or GrK (100 nM, 200 nM) for 24 h. Cells were then incubated with 1∶10 dilution of WST-1 reagent for 3 h and absorbance was read at 450 nM. Cell proliferation was assessed using cell counts. Cells were seeded in 12-well culture dishes and incubated with varying concentrations of GrK for 48 h, with or without ATAP-2 (5 µg/ml). Cells were then trypsinized, centrifuged for 5 min 100× g, re-suspended in 1 ml PBS, diluted 1∶1 in 0.4% Trypan Blue and counted using a hemocytometer. Cells treated with ATAP-2 were incubated for 30 min prior to treatment with GrK.

### Statistical analysis

All data are expressed as mean +/− SEM for three separate experiments. Statistical analysis was performed using One-Way ANOVA followed by Dunnets post hoc analysis for multiple group comparisons. Differences were considered significant at p<0.05.

## Results

### Extracellular GrK is not cytotoxic

**Figure 1:**
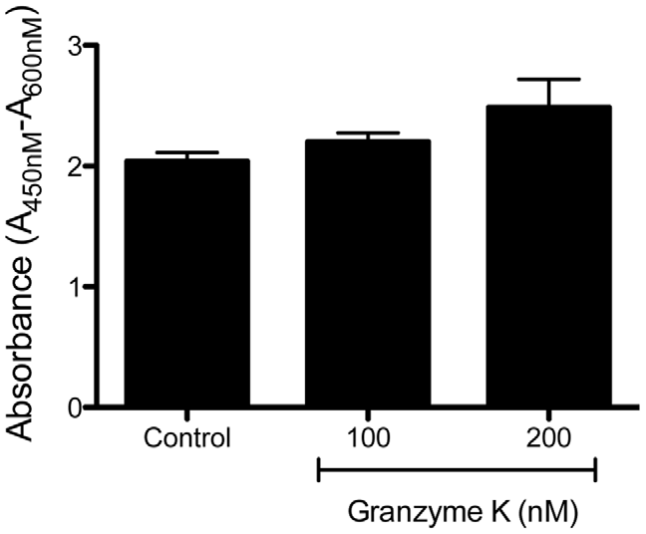
Extracellular GrK is not cytotoxic. HFLs were serum starved and incubated with growth media in the presence or absence of GrK (100 nM, 200 nM) for 24 h and cell viability was assessed using the WST-1 assay. All data are expressed as mean absorbance ± SEM from three independent triplicate experiments.

As extracellular GrA and GrB may induce cell death through the cleavage of extracellular matrix proteins and the subsequent induction of anoikis, we examined the impact of extracellular GrK (in the absence of perforin or any other delivery agent) on cell viability using the WST-1 assay. As demonstrated in Figure 1, HFLs treated with 100 and 200 nM of GrK exhibited similar cell viability to HFLs incubated in media alone (Figure 1).

### GrK induces IL-6, IL-8 and MCP-1 production in HFLs

**Figure 2:**
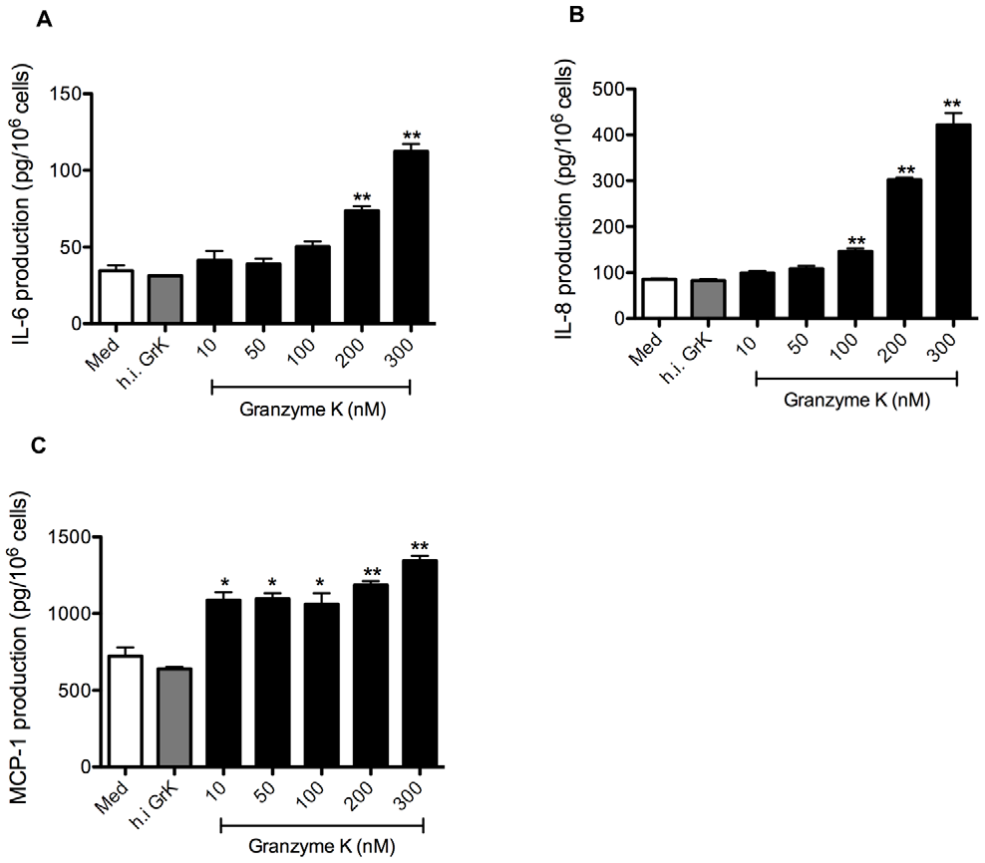
GrK stimulates IL-6, IL-8 and MCP-1 protein production in lung fibroblasts. (A–C) HFLs were exposed to concentrations of GrK ranging from 10–300 nM at for 24 h. Cell culture supernatants were then collected and analyzed for IL-6, IL-8 and MCP-1 production by ELISA. Cell counts were used to normalize cytokine concentrations by controlling for variability in cell numbers. Control wells include cells treated with media alone or cells treated with heat in-activated GrK (100 nM). All data are expressed as mean protein production (pg/10^6^ cells) ± SEM from three independent triplicate experiments. * p<0.05 when compared to media alone; ** p<0.01 when compared to media alone.

HFLs stimulated with GrK at concentrations ranging from 10–300 nM (in the absence of perforin or any other delivery agent) exhibited a dose-dependent increase in IL-6 and IL-8 protein production in cell culture supernatants at 24 h (Figures 2 A and B). Incubation with GrK also induced the secretion of MCP-1 though this release did not appear to be dose-dependent (Figure 2 C). Enzymatic activity was required to stimulate cytokine and chemokine production as heat-inactivated GrK (100 nM) did not induce IL-6, IL-8 or MCP-1 production. Time course experiments using GrK (200 nM) demonstrated that maximal release of IL-6, IL-8 and MCP-1 was detected in supernatants at 24 h (data not shown).

### Induction of IL-6, IL-8 and MCP-1 in HFLs occurs through activation of PAR-1

**Figure 3:**
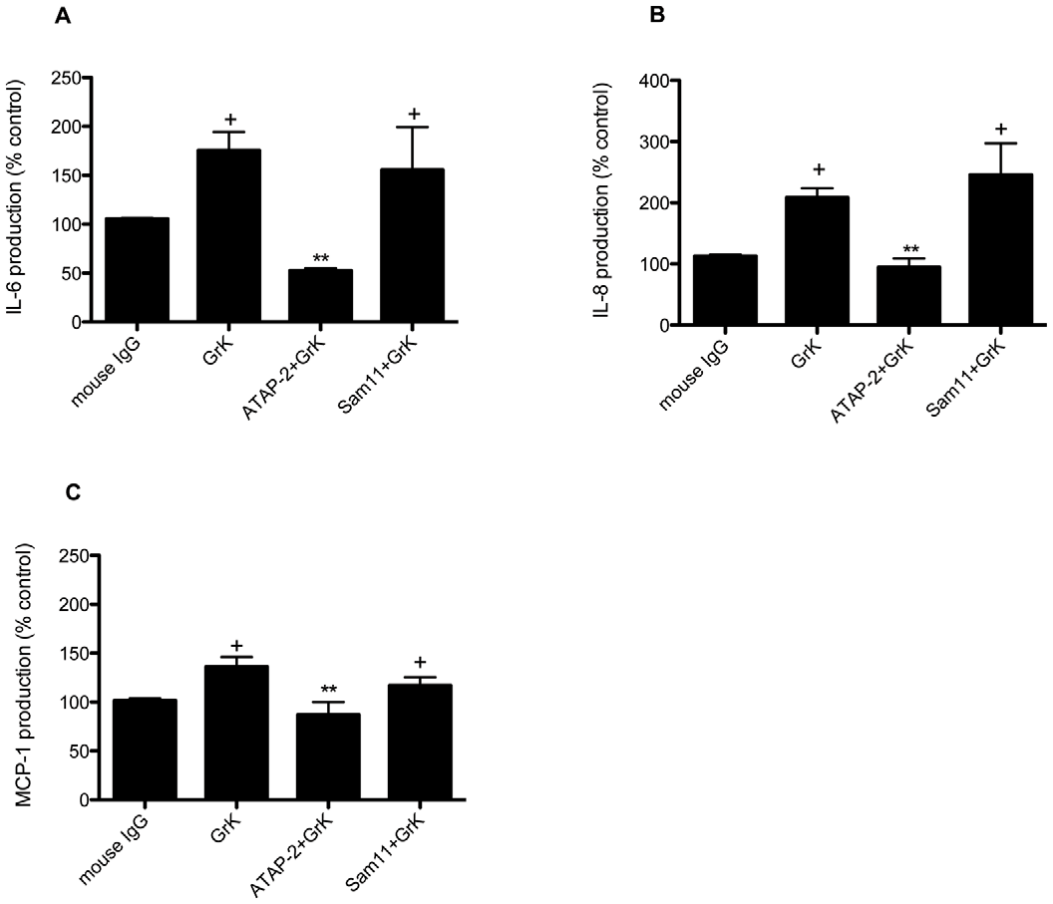
PAR-1 neutralization reduces GrK-induced IL-6, IL-8 and MCP-1 production. Cells were incubated with the PAR-1 neutralizing antibody (ATAP-2; 5 µg/ml) PAR-2 neutralizing antibody (Sam11L; 25 µg/ml) or mouse isotype IgG control antibody for 30 min prior to incubation with GrK (200 nM) for 24 h. Supernatants from cell cultures were collected and analyzed for IL-6, IL-8 and MCP-1 production by ELISA. Cell counts were used to normalized to cell numbers. Data are expressed as % of media control (DMEM+0.0001% NaN_3_) ± SEM from three separate experiments run in triplicate. + p<0.05 when compared to media control; ** p<0.05 when compared to GrK (200 nM) treatment.

Previous studies have established that HFL express PARs-1-4 [30], [31], [32]. Given its trypsin-like activity, we examined whether extracellular GrK was inducing the release of pro-inflammatory molecules through the cleavage and subsequent activation of PARs. To address this, we used antibodies to specifically block PAR-1 and PAR-2 cleavage sites, using a PAR-1 neutralizing antibody as described previously [33], [34], [35]. As shown in Figure 3 A–C, incubation with the PAR-1 neutralizing antibody (ATAP-2) prior to GrK treatment markedly diminished IL-6, IL-8 and MCP-1 production, whereas incubation with the PAR-2 (Sam11L) neutralizing antibody had no significant impact on these events. In the current study, we did not specifically neutralize PAR-3 or PAR-4 because we observed no cytokine production following treatment with the potent PAR-4 agonist peptide GYPGQV (data not shown). While PAR-3 and PAR-4 can be proteolytically activated by thrombin, evidence suggests that PAR-3 acts as a cofactor for PAR-4 at low concentrations of thrombin and is a non-signaling receptor [36]. Furthermore, we observed near complete ablation of cytokine production using the PAR-1 neutralizing antibody suggesting from a functional standpoint that most of the biological activity observed following GrK treatment is the result of PAR-1 activation, but does not completely rule out a potential role for PAR-3 or PAR-4.

### PAR-1 receptor desensitization reduces GrK-induced IL-6, IL-8 and MCP-1 production

**Figure 4:**
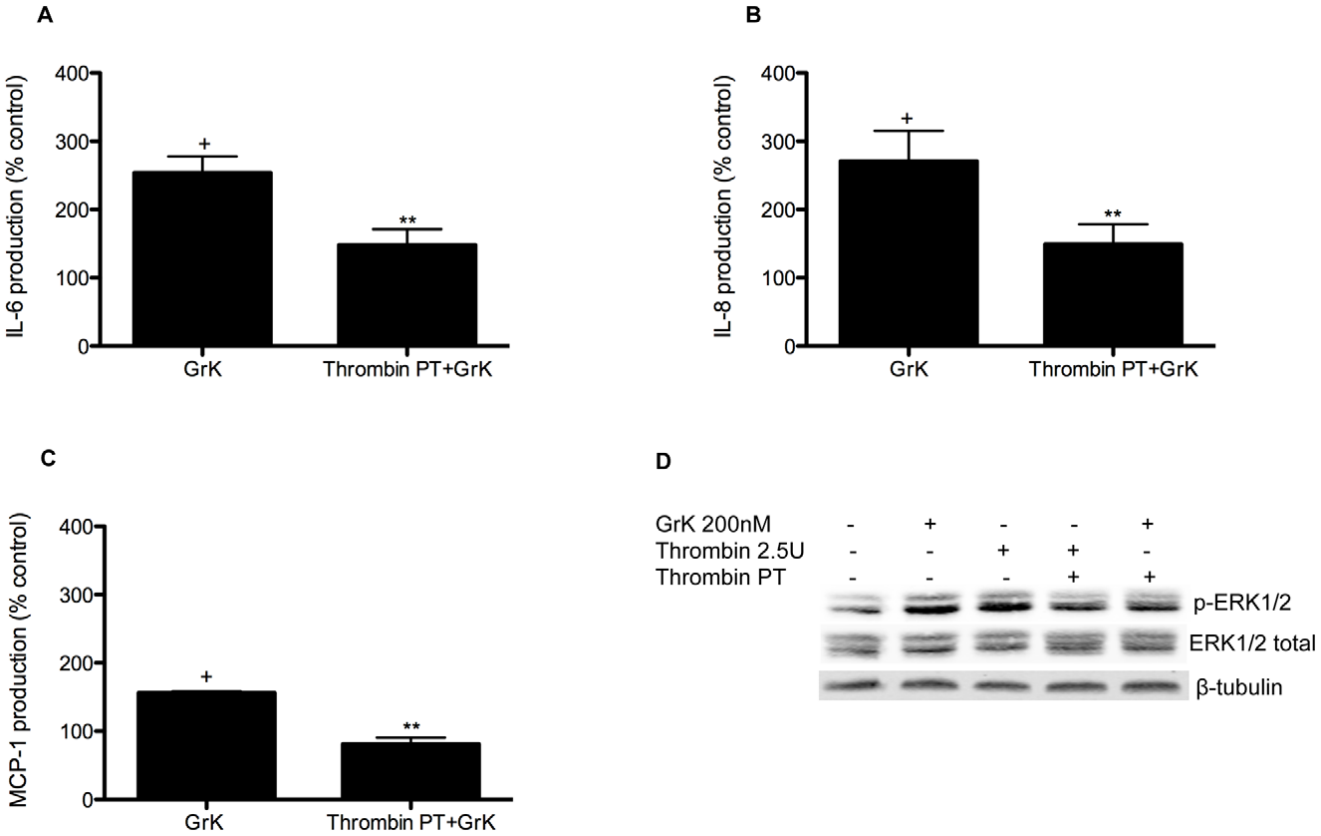
Desensitization of PAR-1 with Thrombin (2.5 U/ml) reduces GrK-induced IL-6, IL-8 and MCP-1 production in lung fibroblasts. (A–C) HFL were treated with thrombin (2.5 U/ml) for 10 min, monolayers were then rinsed three times with fresh media and incubated with GrK (200 nM) for 24 h. Supernatants were collected and IL-6, IL-8 and MCP-1 levels were analyzed by ELISA. Supernatants were normalized using cell counts. Data are expressed as % change over media control ± SEM from three separate experiments run in triplicate. (D) GrK (200 nM) and thrombin (2.5 U/ml) induced ERK1/2 phosphorylation following 10 min incubation. Pre-treatment (PT) of HFL with thrombin (2.5 U/ml) for 10 min prior to treatment with GrK (200 nM) for an additional 10 min decreased ERK1/2 phosphorylation when compared to monolayers incubated with GrK (200 nM) alone. ERK1/2 phosphorylation was assessed by Western blotting of cell lysates using anti-phospho-ERK1/2 and anti-total-ERK1/2 antibodies. Protein loading was verified and normalized using β-tubulin. Data represent the mean ± SEM of at least three independent experiments. + p<0.05 when compared to media control (1% DMSO in DMEM); ** p<0.05 compared to GrK (200 nM) treated cells.

To further verify the specificity of GrK-mediated PAR-1 activation, we used the high affinity ligand for PAR-1, thrombin (2.5 U/ml) to desensitize PAR-1 prior to exposure to GrK. As shown in Figure 4 (A–C), an initial proteolytic activation of PAR-1 by thrombin prior to incubation with GrK decreased IL-6, IL-8 and MCP-1 production. This was further confirmed by examination of ERK1/2 phosphorylation where pre-treatment (PT) with thrombin decreased the level of phosphorylation elicited by subsequent application of GrK (Figure 4 D).

### GrK induces ERK1/2 and p38 MAPK phosphorylation in HFL

**Figure 5:**
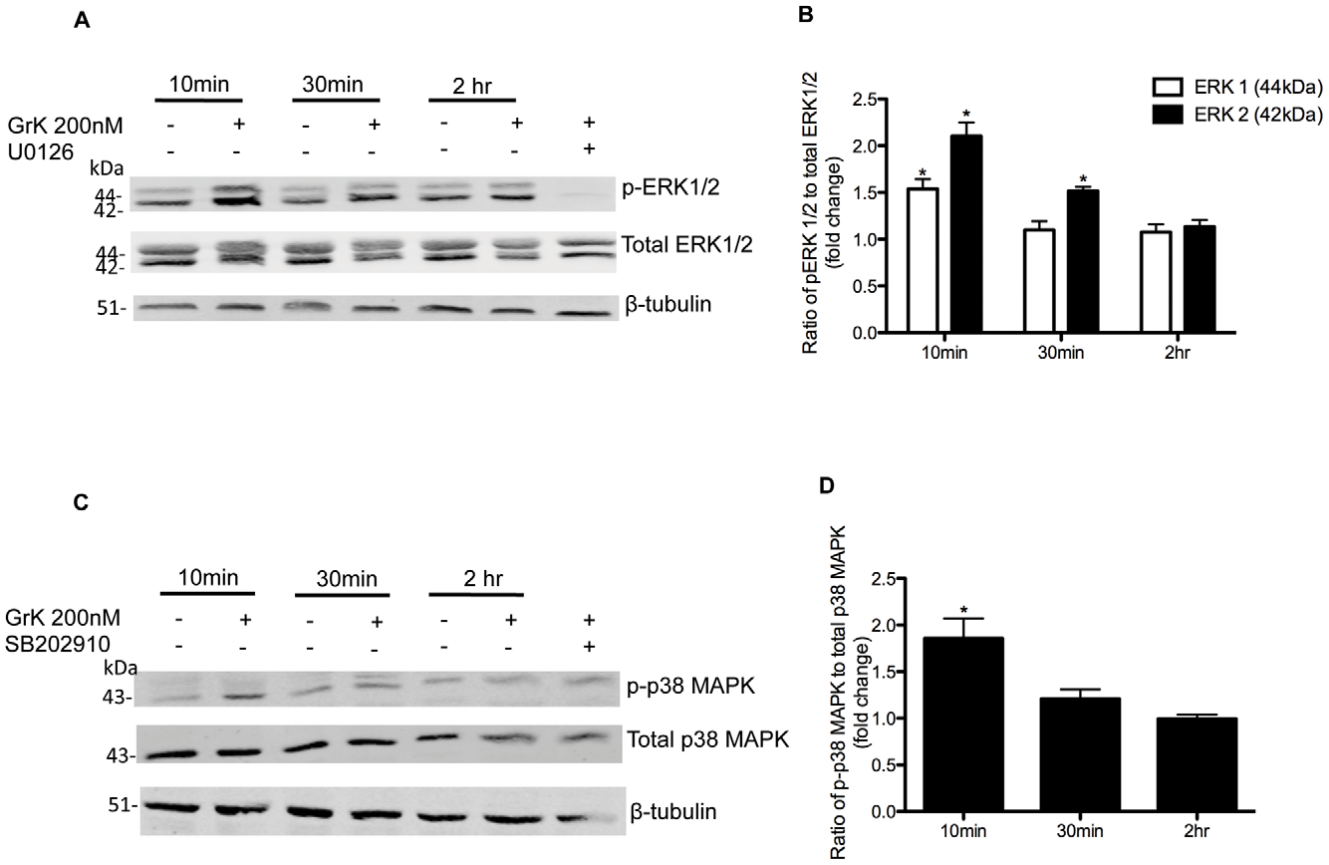
Western blot analysis of effect of GrK on ERK1/2 and p38 MAPK phosphorylation. Cells were incubated with GrK (200 nM) for 10 min, 30 min, or 2 h. Cell lysates were collected and screened for (A) ERK1/2 phosphorylation using anti-phospho-ERK1/2 and anti-total-ERK1/2 antibodies and (C) p38 MAPK phosphorylation using anti-phospho-p38MAPK and total p38MAPK antibodies. Cells incubated with ERK1/2 inhibitor (U0126; 10 µM) or p38MAPK inhibitor (SB202190; 10 µM) prior to treatment with GrK (200 nM) for 10 min are included to demonstrate the activity of each inhibitor. Densitometry analysis of immunoblots was carried out using Li-COR Odyssey Infrared imaging system (Li-COR biosciences). (B) Relative levels of ERK1/2 phosphorylation (ERK1 white bars; ERK2 black bars) are expressed as a ratio of phospho-ERK1/2 to total ERK1/2. (D) Relative levels of p38 MAPK phosphorylation are expressed as a ratio of phospho-p38 MAPK to total p38 MAPK. Protein loading was normalized using β-tubulin. The values shown are mean +/− SEM from three separate experiments. * p<0.05 compared to media alone.

It has previously been reported that activation of PAR-1 leads to the activation of ERK1/2 and p38 MAPK in several cell types, including fibroblasts [37], [38], [39]. Thus, we next performed experiments to determine if GrK activation of PAR-1 induced ERK1/2 or p38MAPK phosphorylation in HFL. Figure 4 A–D demonstrates that incubation with GrK (200 nM) induced phosphorylation of both ERK1/2 and p38MAPK. Phosphorylation of ERK1/2 was maximal 10 min after stimulation with GrK and gradually declined to control levels by 2 h incubation (Figure 5 A and B). GrK also induced p38 MAPK phosphorylation with a similar kinetic profile (Figure 5 C and D).

### MAPK activation in PAR-mediated IL-6, IL-8 and MCP-1 release

**Figure 6:**
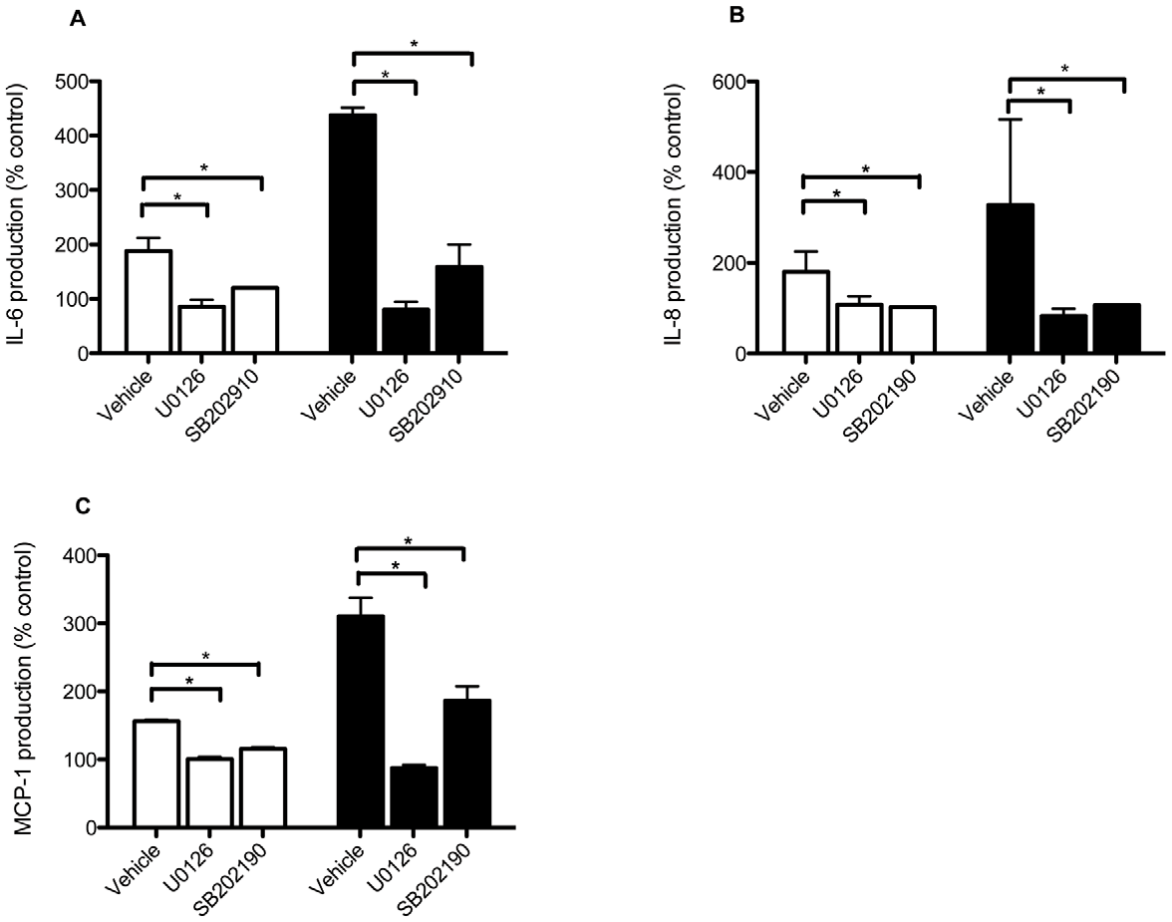
ERK1/2 is required for GrK-induced IL-6 and IL-8 production in lung fibroblasts. The effect of U0126 or SB202910 on IL-6, IL-8 and MCP-1 production following GrK (white bars) or thrombin (black bars) treatment. Cells were treated with 10 µM U0126 or 10 µM SB202910 for 45 min prior to exposure to GrK (200 nM) or thrombin (2.5 U/ml) for 24 h. Supernatants were collected and analyzed for protein production by ELISA. Data are expressed as percent change over vehicle control (1% DMSO in DMEM) ± SEM from experiments run in triplicate. * p<0.05 when compared to GrK or thrombin treatment alone.

Using specific pharmacological inhibitors, we assessed the relative roles of ERK1/2 and p38 MAPK activation on the synthesis and release of GrK- and thrombin-induced IL-6, IL-8 and MCP-1 in HFL. As shown in Figure 5, pre-treatment of cells with the MEK1/2 inhibitor U0126 inhibited GrK-induced IL-6 and IL-8 release, and thrombin-induced IL-6, IL-8 and MCP-1 release back to control levels. While a reduction was noted in GrK-induced MCP-1 levels, levels were not significantly different from controls (Figure 6 C). In contrast, inhibition of p38 MAPK with SB202910 (10 µM) had no significant impact on GrK-induced IL-6, IL-8 or MCP-1 production but did significantly reduce thrombin-induced IL-6, IL-8 and MCP-1 levels, suggesting that although GrK can activate p38 MAPKs in HFL, p38 may not be involved in mediating PAR-1 induced IL-6, IL-8 or MCP-1 production.

### GrK stimulates HFL proliferation

**Figure 7:**
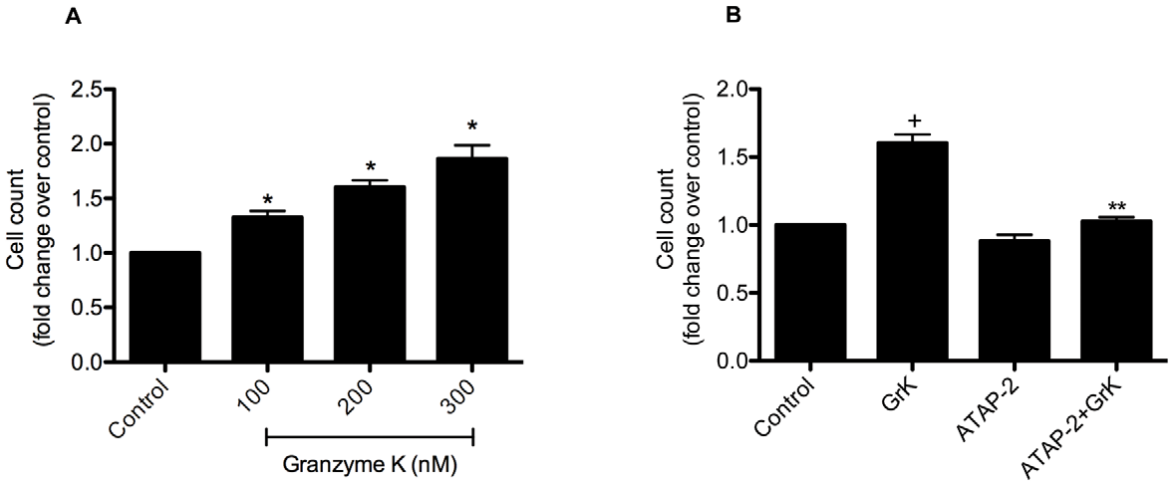
GrK induces cell proliferation in HFLs in a dose-dependent manner. (A) Cells were incubated with 100 and 200 nM of GrK for 48 h then trypsinized and counted using a hemocytometer to determine cell number. (B) PAR-1 neutralization with ATAP-2 (5 µg/ml) abolished GrK (200 nM)-induced cell proliferation. Data are expressed as mean fold change ± SEM from three independent experiments run in triplicate. * p<0.05 compared to media control.

Because fibroblasts are a major component of connective tissues and fibroblasts proliferation is an important process in wound healing, we examined the functional consequences of GrK-induced signaling on fibroblast viability and proliferation. As can be seen in Figure 7, GrK stimulated proliferation in a concentration dependent manner (A). No significant difference was detected between controls and GrK-treated cells prior to 48 h (data not shown). GrK (200 nM)-induced proliferation was inhibited by ATAP-2 (5 µg/ml) (black bars) suggesting that GrK-induced proliferation is occurring through a PAR-1 mediated mechanism (B).

## Discussion

In this study we demonstrate that GrK can induce pro-inflammatory cytokine release from human lung fibroblasts through the activation of PAR-1 and supports previous studies that have suggested that GrK is not involved in immune-mediated cytotoxity but rather in the promotion of inflammation [3], [7], [40], [41]. Previous work by Joeckel *et al* [7] demonstrated that GrK induces IL-1β production and secretion from mouse peritoneal macrophages. The IL-1β processing observed in this study was dependent on both the concentration of GrK and its delivery into cells by streptolysin O (SLO). Interestingly, the authors noted that high concentrations of GrK (600–1000 nM) resulted in IL-1β production in the absence of SLO. Whether GrK was exerting this effect via an extracellular or intracellular mechanism was not established. Our study not only demonstrates that GrK can promote inflammation through IL-6, IL-8 and MCP-1 production but also provides evidence that GrK can act extracellularly to exert this function in the absence of a cell delivery agent. Given the tryptase-like activity of GrK, and the role that PARs play in inflammation, it is not unreasonable to hypothesize that in conditions of elevated, free GrK it could cleave and activate PAR-1 and promote inflammation.

PAR-1 is involved in a variety of biological events and is the primary receptor responsible for mediating many of the pro-inflammatory and pro-fibrotic effects of thrombin [38], [39], [42]. PAR-1 activation triggers the production of several inflammatory mediators, including IL-6, IL-8 and MCP-1, and induces cell proliferation through the coupling of receptor activation to one or more intracellular signaling pathways [26], [27]. In the present work, neutralizing antibodies and receptor desensitization experiments suggest that GrK induced IL-6, IL-8 and MCP-1 production is dependent on PAR-1 activation through a pathway involving ERK1/2 and p38 MAPK. Furthermore, as is seen with thrombin, GrK-induced IL-6, IL-8 and MCP-1 production is also dependent on ERK1/2 and p38MAPK activation [38], [39]. While we focused on ERK1/2 and p38 MAPK signaling in this study, GCPRs can also initiate other signaling cascades pathways (reviewed in [26], [27], [29]). Recently, Deng et al (2008) demonstrated that MCP-1 release in murine lung fibroblasts was regulated by multiple pathways including phospholipase C, calcium-dependent PKC, and Rho kinase signaling pathways [37]. GrK-induced PAR-1 activation is likely associated with multiple signaling pathways and further biochemical analysis is required to delineate which signaling pathways are involved.

Although several clinical studies have observed elevated extracellular GrK in disease, this is the first study to demonstrate a physiological role for extracellular GrK in cytokine production. The observation that GrK can initiate cytokine release and induce cell proliferation in lung fibroblasts is significant given recent observations that extracellular GrK is detected in the BAL of patients with viral pneumonia and allergic asthma [10], [11]. IL-6, IL-8 and MCP-1 are important mediators in the regulation of the acute-phase response to injury and infection by influencing immune cell recruitment, as well as the differentiation and activation of T cells and macrophages [43], [44], [45]. In the current study, elevated levels of MCP-1 were detected in supernatants following treatment with low concentrations of GrK. MCP-1 is expressed by numerous cell types, including monocytes/macrophages, fibroblasts, and epithelial cells, and is a potent chemoattractant for mononuclear cells. In addition to its ability to promote inflammation, MCP-1 is involved in the recruitment of fibrocytes, and may exert profibrotic effects by inducing the expression of transforming growth factor-β and by down-regulating the production of the major anti-fibrotic prostaglandin E_2_ [46], [47], [48]. Interestingly, elevated levels of MCP-1 are correlated with poor outcomes in patients with interstitial lung disease, suggesting that GrK may not only influence inflammation, but may also be involved in tissue repair and airway remodeling [46], [49], [50], [51], [52]. However, whether or not GrK plays a direct role in the pathogenesis of such diseases will require the use of GrK-knockout animals that are unavailable at present.

While proteases can positively impact inflammation, chronically elevated levels of proteases, particularly in the absence of the appropriate anti-protease, can be detrimental.

In cases of sepsis, elevated levels of free, active GrK and decreased levels of IAIP correlate with the severity of sepsis. Elevated levels of extracellular GrK, particularly in the absence of IAIP, may contribute to the enhanced expression IL-6, IL-8 and MCP-1 in such conditions. Given that sepsis and pneumonia are common causes of acute lung injury leading to inflammation and fibrosis [17], future studies are necessary to determine whether a causal role of GrK in such diseases exists.

In summary, in the present study we demonstrate that extracellular GrK is capable of inducing cytokine production from human lung fibroblasts through the activation of PAR-1. Future studies are required to fully understand the extracellular roles of GrK on structural cells within the lung, we well as other tissues, with the potential that GrK could be targeted therapeutically as a means of reducing PAR-1 mediated inflammation.
